# Milk allowance affects play responsiveness in calves

**DOI:** 10.1017/awf.2026.10091

**Published:** 2026-07-15

**Authors:** Amelia St John Wallis, Michael T. Mendl, Benjamin Lecorps, Suzanne D.E. Held

**Affiliations:** Bristol Veterinary School, University of Bristol, United Kingdom

**Keywords:** Animal behaviour, animal well-being, cattle, milk restriction, play contagion, positive animal welfare

## Abstract

Play behaviour is increasingly being used as an animal welfare indicator. Calves on standard milk allowances, thought to be experiencing hunger, often play less than those fed more due to lower nutrition, negative affect, or both. However, various aspects of play can be measured, and the most sensitive forms of play to hunger and welfare are unclear. We explored the relationship between milk allowance and various play behaviours and characteristics, including those that have received less attention, such as play responsiveness (i.e. the likelihood of a calf playing in response to their partner’s locomotor play). Pair-housed calves (n = 22) on a commercial dairy farm were provided either 6 L milk per day (standard) or 12 L milk per day (enhanced). Each pair included one calf from each diet group. At 16 days old (7 days after imposition of the diets), the calves were habituated to the play arena, before their play behaviours were assessed firstly in pairs and then individually on two consecutive days. Unexpectedly, milk allowances did not affect locomotor play (i.e. running, kicks/bucks) in either test. The expected effects may have been masked by the calves’ high motivation to play in the arena, as their home pens greatly limited play opportunities. However, calves fed less milk showed lower play responsiveness, potentially due to them having a higher ‘threshold’ for play being elicited or having reduced motivation to engage in social play. The group differences in play responsiveness suggest that this variable may be a more sensitive welfare indicator warranting further research.

## Introduction

In non-human animals (hereafter referred to as ‘animals’), recent conceptualisations of welfare focus on the “*balance of positive and negative affective states*” of the individual (Reimert *et al.* 2013). In this context, play behaviours have commonly been used as an indicator of affect and welfare (for reviews, see Held and Špinka 2011; Ahloy-Dallaire *et al.* 2018; St John Wallis *et al.* 2025b), as play behaviours tend to be suppressed in response to situations that challenge fitness and cause stress (Lawrence 1987; Fraser & Duncan 1998; Burghardt 2005; Bateson & Martin 2013). On the other hand, higher levels of play behaviour are thought to indicate the presence of positive affect, making play behaviour a potential positive welfare indicator (Held & Špinka 2011; Ahloy-Dallaire *et al.* 2018). However, play behaviour is complex, encompassing both individual and social elements in many species (e.g. calves: Jensen & Kyhn 2000), and the optimal way to use play behaviour as a welfare indicator is not yet clear.

One context in which play behaviour has been used to reflect changes in affective state and welfare is in relation to feed restriction in pre-weaned calves. Standard milk allowances (approximately 6 L per day; Costa *et al.* 2019) are significantly lower than a calf’s *ad libitum* milk intake (i.e. approximately 10–13 L per day across studies: Jasper & Weary 2002; Frieten *et al.* 2017; Welboren *et al.* 2019), and multiple studies have reported that calves fed less milk also play less. For example, in studies measuring home pen play behaviour, single-housed calves fed 5 L of milk per day compared to 9 L per day played less following straw provision, combining social, locomotor, and object play (e.g. butting another calf, galloping/trotting, and playing with straw; Duve *et al.* 2012). Standard-fed calves also engaged in less locomotor play (i.e. galloping, leaping, turning, and bucking) than those fed enhanced milk allowances, and the group differences were eliminated when the enhanced-fed calves were returned to the standard diet (Jensen *et al.* 2015). Finally, Krachun *et al.* (2010) reported that calves fed 6 L of milk per day vs 12 L engaged in less running (i.e. galloping and trotting) in the home pen at 3 weeks of age, correlating with their energy intake. Similar findings have also been reported when using arena tests, where calves are placed into an arena larger than their home pen to stimulate play behaviour (Mintline *et al.* 2012). For example, Rushen and de Passillé (2012) reported that calves engaged in less running at weaning, when their milk allowance is removed, compared to the week before in an individual arena test. However, another study unexpectedly showed that calves fed only 75% of the standard allowance engaged in more jumping and running at 6 weeks of age than those fed 100% of it (Rushen & de Passillé 2014).

On balance, play levels generally appear to be reduced by feed restriction in calves, potentially reflecting hunger (i.e. “*a negative subjective state experienced by an animal that is chronically undernourished*”: D’Eath *et al.* 2009) and lower nutrition levels (Krachun *et al.* 2010; Rushen & de Passillé 2012), as well as negative affect, as hunger negatively affects mood in humans (MacCormack & Lindquist 2019). However, there is more evidence focusing on locomotor play duration than on other aspects of play, such as social behaviours. Calves are highly social, as indicated by their motivation for social contact (Holm *et al.* 2002; Ede *et al.* 2022), and engage in social forms of play, such as play fighting (Jensen *et al.* 2015) and running with other calves (parallel locomotor play: Jensen *et al.* 2015; Bailly-Caumette *et al.* 2023).

Play behaviour is also highly contagious (Bekoff 2001), whereby the behaviour spreads from one individual to another, as shown experimentally in rats (Pellis & McKenna 1992) and pigs (Reimert *et al.* 2013). One study reported that calves on high milk allowances housed with others on low allowances played less than calves in all-high allowance groups, suggesting an effect of feed restriction on the group’s play dynamics through what the authors termed “*negative play contagion*”, defined as “*the suppression of play behaviour by conspecifics*”, whereby one calf’s play levels are negatively affected by the low play levels of others within the group (Größbacher *et al.* 2020). The way in which feed restriction drives these changes in group play dynamics is worthy of further exploration, as such effects may provide an interesting avenue for understanding how different aspects of play behaviour reflect the individuals’ experiences.

This work aimed to explore how standard vs enhanced milk allowances (i.e. 6 L of milk per day vs 12 L) affect different aspects of play behaviour in calves using two arena tests (paired and individual arena tests), allowing for the identification of both individual and social play variables. We predicted that, compared to enhanced-fed calves, those in the standard-fed group would play less in the arena tests and potentially be less responsive to play contagion and, thus, less likely to engage in play behaviour when their companion started playing. We assumed that the two diet groups would generate a *relative* difference in hunger and, thus, affective state. However, it is unclear whether either group can be considered to represent a ‘neutral’ level of affect on a continuum from more negative to more positive affective state. As such, any effects on play behaviour could be driven by more negative affect resulting from hunger in the standard-fed group and/or more positive affect in the enhanced-fed group due to being fed more and experiencing satiety.

## Materials and methods

### Ethics statement

This work was approved by the University of Bristol’s Animal Welfare and Ethics Review Board (AWERB; UIN-23-075 and UIN-22-020). The number of animals used for research was minimised by conducting this study with calves enrolled in a separate approved project investigating the effect of standard vs enhanced milk allowances on cognition. Calves were habituated to the play arena in their home pen pairs prior to testing and had a minimum break of 2 h between their cognitive testing at morning feeding (which lasted for a maximum of 5 min) and the play tests. No calves were feed restricted for the purposes of this study, as the treatment group received more milk than standard at the farm.

## Study animals and housing

This study was conducted at Wyndhurst Farm, University of Bristol, UK between February 2024 and August 2024. In line with standard procedures at the farm, the calves were fed two meals of colostrum within 24 h after birth, housed as a pair in straw-bedded hutches with 2.5 × 1.5 m (length × width) of indoor hutch space and a 1.6 × 1.5 m fenced outdoor area, and given *ad libitum* access to water in a bucket filled by hand when required, as well as grain (18% Premium Calf Feed Blend, Tamar Milling, Whitstone, UK) and straw in two separate feeders that were filled daily. The calves were fed 6 L of milk per day made from milk powder (Sprint Plus 50, Bridgmans Farm Direct, Shepton Mallet, UK; 18% crude fat, 22% crude protein) mixed at 190 g powder per L. This mixing composition provides approximately 3.4% fat and 4.2% protein, whereas whole milk provides approximately 3.6% fat and 3.4% protein (Public Health England 2015). Half of the allowance was fed in the morning (at approximately 0800h) and half in the evening (at approximately 1600h).

At 9.7 (± 2.1) days old, n = 22 calves (18 beef cross [6 male and 12 female], 4 Holstein female) were enrolled in the study (for power calculations, see *Statistical analysis*). Prior to enrolment, the calves were housed into pairs by the farm staff according to their age and breed (i.e. with calves born at similar times and of the same breed generally housed together), and the pairs were kept the same for the study. In each enrolled pair, one calf was allocated to receive 6 L of milk per day (standard feed group), and the other was allocated to receive 12 L (enhanced feed group). Calves in the pairs were allocated to the treatment groups based on birth sequence in an alternating manner as each subsequent pair was enrolled. The two milk allowances are referred to as the standard vs enhanced feed groups, as calves are often provided with 6 L of milk per day or less as part of routine management on dairy farms (Costa *et al.* 2019). On average, the calves in the standard feed group drank 5.9 (± 0.05) L of milk per day, and those in the enhanced feed group drank 9.5 (± 0.68) L of milk per day during the study. As such, the enhanced feed group drank, on average, 3.6 L more milk than the standard feed group, representing a 61% higher milk intake. At 1 week after their enrolment, the calves began the play testing (average age = 16.7 [± 2.1] days old). No calves had to be excluded due to sickness, with the assessment of this based on the presence of scouring, elevated temperature, and/or not wanting to drink milk meals.

## Play arena exposure

**Figure 1.**
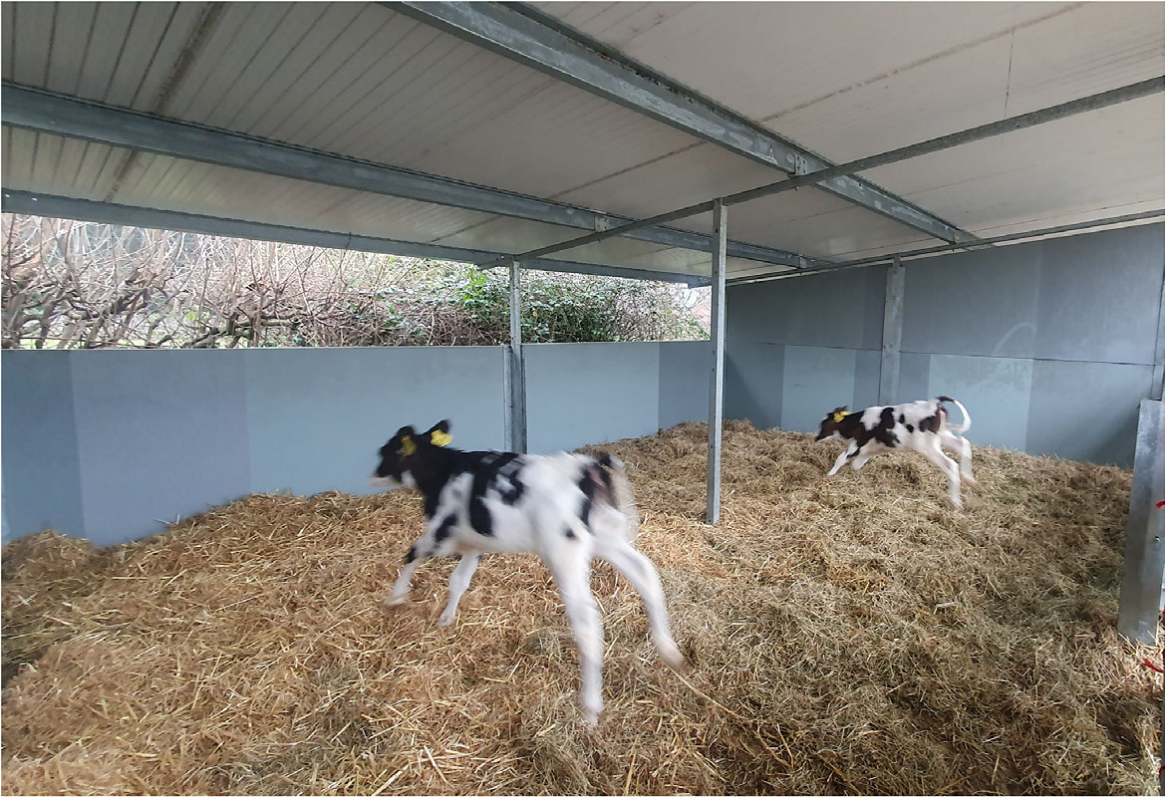
Two calves play during the paired test. Calves were pair-tested with their familiar pen-mate (one from each diet group per pair) and allowed to play in the play arena for 15 min.

On Day 1 of the play testing procedure, the calves were led in their pairs to a straw-bedded arena measuring 6.3 × 4.93 m (length × width; Figure 1) and allowed to remain there for 10 min to habituate to this new environment. On Day 2, the calves were led to the arena in their pairs (pair test) for 15 min. On Day 3, the calves were led to the arena individually for 15 min (individual test). Play arena habituation and testing was conducted between 1200 and 1300h (i.e. 4–5 h after morning feeding).

The arena test was used as the small sizes of the home pens (i.e. 1.2 m^2^ of outdoor space and 1.9 m^2^ of indoor space per calf, separated by a narrow hutch aperture) restricted opportunities to engage in play behaviour. However, when using the arena test, it should be noted that play levels are not independent of the home pen environment. For example, motivation to engage in locomotor play in a larger play arena was found to be higher in calves housed in more restrictive home pens (Jensen & Kyhn 2000) and following a period of deprivation (Jensen 2001). In this work, play arena testing was conducted daily and with an initial habituation session to reduce this motivational rebound.

### Behavioural observations

Play behaviour in the tests was recorded using a GoPro 11 (GoPro Inc, CA, USA), and behavioural observations were recorded using BORIS software (version 8.25.4). The primary observer, blind to treatment group, recorded play behaviour for all of the calves. A secondary observer, also blind to treatment group, recorded play behaviour for eight calves across five videos (three paired tests with two calves each; two individual tests) to calculate inter-observer reliability scores.

The ethogram is presented in Table 1. Running (which included galloping, cantering, and trotting) and bucking and kicking were defined based on previous work in calves by Mintline *et al.* (2012). Based on the work of Krachun *et al.* (2010) and Mintline *et al.* (2012), running bouts were defined by events separated by 3 s or more. For the paired arena test only, play-fighting was described as calves pushing (butting) their heads against each other or one calf pushing their head against the other, based on the definition of Jensen and Kyhn (2000). Since few instances of social play-fighting were observed, this variable was not included in the analysis. Finally, instances of initiating vs joining in with the running bouts of the other calf were recorded (not previously assessed in the calf literature to our knowledge). For instance, when one calf started to run and was joined by the other calf either during the running bout or within 3 s after, play initiation was coded for the first calf and play joining for the second calf. Video examples of a calf joining in and not joining in when its companion engages in locomotor play can be seen as mp4 files, ‘Example of play joining’ and ‘Example of not joining’, in the Supplementary material.

**Table 1.** Ethogram of play behaviour in calves. Table 1. long description.

Behaviour	Description
Running	The calf trots, canters, or gallops. Run bouts separated by 3 s or more are counted as two different bouts. If the pause in running is less than 3 s, state recording continues.^1^
Buck	The calf kicks both of its hind legs up or back.^1^
Kick	The calf kicks one of its hind legs up, back, or sideways.^1^
Play fighting	Two calves push their heads against each other, or one calf pushes its head against the other calf.^2^
Play initiation and play joining (paired test)	One calf is running (i.e. the initiator), and the other calf starts to run at the same time (i.e. the joiner). These behaviours are also coded if one calf starts running within 3 s of the other calf finishing their running bout.

The footnotes indicate the papers which certain descriptions were based on 1: Mintline *et al.* (2012); 2: Jensen and Kyhn (2000).

### Inter-observer reliability

Reliability scores between the two raters were excellent for all behaviours (run duration ICC = 0.997; run bouts ICC = 0.987; point play ICC = 0.981; play initiation/joining ICC = 0.957).

### Statistical analysis

A power analysis was conducted in advance based on Größbacher *et al.* (2020), who compared play behaviour in calves in three groups: mixed feed levels (i.e. a mix of calves on low and high milk allowances); all calves on a high milk allowance; and all calves on a low milk allowance. For identifying an effect of having mixed feed levels vs all high milk allowances on locomotor play, the effect size was estimated to be d = 1.74 (α = 0.05, β = 0.80) for run duration and d = 1.49 for run bout frequency, giving required sample sizes of n = 14 and n = 18, respectively. However, this previous study used much longer home pen observations over 18 h and conducted analyses at the group level (groups of 3), whereas our work here aimed to analyse individual behaviour within calf pairs, and no previous studies have investigated the effect of feed restriction on the initiation and joining of locomotor play bouts in a paired arena test. Therefore, here, we used a slightly larger sample of n = 22 calves.

All analyses were conducted using IBM SPSS® Statistics (Version 29.0.2.0). The dataset and SPSS syntax are provided in the Supplementary material. To explore the effect of milk allowances (standard vs enhanced feed groups; n = 11 per group) on play behaviour outcomes, generalised linear mixed models were performed using the identity link function based on the use of continuous outcome variables. Visual inspection of the residual vs predicted plots for non-random patterns (e.g. systematic curvature, funnel-shaped variance indicative of heteroscedasticity, clustering, outliers) was used for model diagnostics, and no substantial deviations from model assumptions were observed. In these models, each calf was treated as the statistical unit, as the calves in each pair were assigned to different milk allowances. For all of the models, calf ID within pair was included as a random effect, and feed group (i.e. standard vs enhanced feed) was included as a fixed effect. To ensure model stability, breed was not included due to the imbalance in breed numbers (i.e. n = 4 Holstein vs n = 18 beef cross) and the fact that the Holstein calves were housed within two pairs. The GLMMs were run using the following outcome variables (giving seven models in total): individual arena test running duration; individual arena test running bout frequency; individual arena test ‘point play’ frequency (i.e. the number of bucks and kicks); paired arena test running duration; paired arena test running bout frequency; paired arena test point play frequency; and paired arena test play responsiveness (%).

Play responsiveness (%) was calculated as the percentage of running bouts initiated by the companion calf that the subject calf joined, thus controlling for the number of opportunities to join a play bout that the subject calf experienced. It should be noted that if the companion calf played very little, this would significantly reduce the subject calf’s responding opportunities. However, the calves in the enhanced and standard feed groups engaged in an average of 21 (± 7.6) and 18 (± 6.0) locomotor play bouts, respectively, in the paired arena test, and the number of locomotor play bouts did not differ significantly between the groups (see *Results*).

## Results

**Figure 2.**
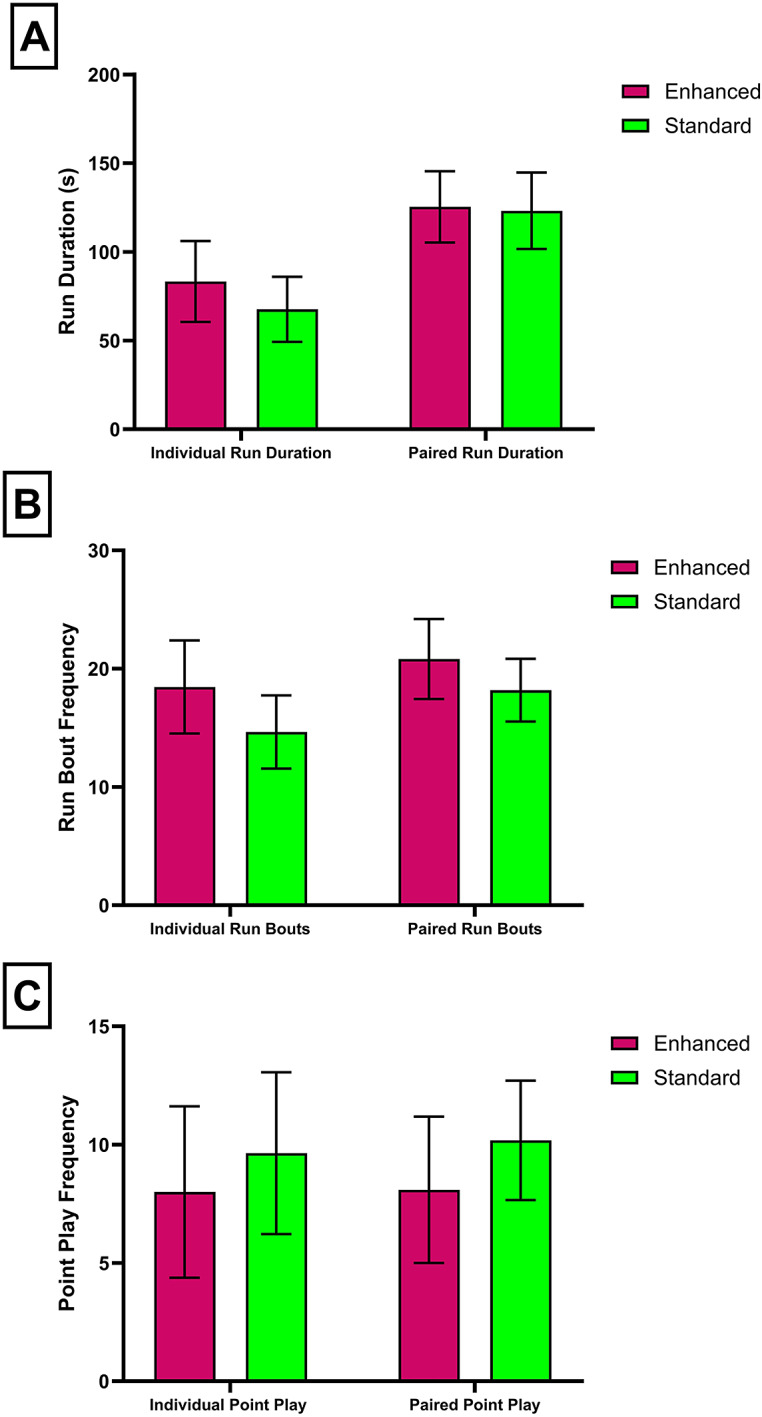
Locomotor play behaviours, including (A) run duration, (B) run bouts, and (C) point play, in the individual and paired arena tests for the standard-fed (6 L per day) and enhanced-fed (up to 12 L per day) calves (n = 22). Calves were given 15 min to play in a play arena either in pairs of familiar calves or alone. Error bars = 95% CI. Figure 2. long description.

**Figure 3.**
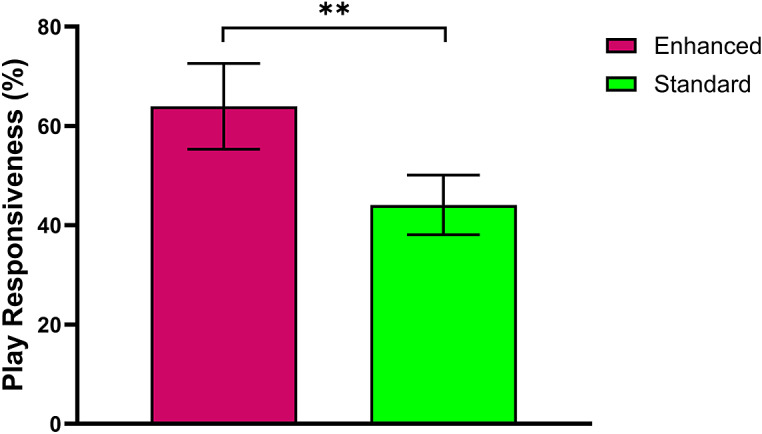
Play responsiveness (as % of responding opportunity based on the locomotor play bouts initiated by the companion calf) for the standard-fed (6 L per day) and enhanced-fed (up to 12 L per day) calves (n = 22) in the paired arena test. Calves were given 15 min to play in pairs of familiar calves. Notes: Error bars = 95% CI; ** *P* < 0.01.

Milk allowances did not affect calves’ locomotor play behaviours (i.e. run duration, run bout frequency, and point play frequency) in the paired or individual arena tests (all *P*-values > 0.05; see Figure 2). However, in the paired arena test, the standard-fed calves showed lower play responsiveness (play responsiveness mean difference = −19.86%, SE = 6.32; F(1, 20) = 9.88; *P* = 0.005; Figure 3), despite the total number of running bouts engaged in by the calves in the two groups not being significantly different. Overall, in the paired test, the standard-fed calves were less likely to join in with the locomotor play bouts of their companion calf compared to the enhanced-fed calves and *vice versa.*

## Discussion

The aim of this work was to further explore the use of play behaviour as a welfare indicator by assessing how milk allowances affect both (i) calves’ locomotor play and (ii) their responses to locomotor play in their companion in an arena test. Our results showed that locomotor play behaviours did not differ between treatments. However, standard-fed calves were less responsive to play contagion, meaning they were less likely to join in with locomotor play in response to their companion calf playing.

There was no effect of milk allowance on the locomotor play variables of run duration, run bouts, and point play. Although this finding could suggest that the different milk allowances did not have the expected effects on mood or energy balance, the calves in the enhanced feed group drank on average 3.6 L more milk per day (i.e. 61% more milk) than those in the standard feed group; as such, it is more likely that locomotor play in the arena was not a sufficiently sensitive indicator of the calves’ experiences of the milk allowances in this study. Indeed, multiple previous studies have shown reduced locomotor play in calves fed less milk when energy intakes are reduced (Krachun *et al.* 2010; Rushen & de Passillé 2012), and following separation from the dam in calves not previously habituated to automated milk feeders (Rushen *et al.* 2016), with this reduction in play behaviour correlating with lower energy intake (Rushen *et al.* 2016).

Calves in the previous studies were observed in their home pens rather than tested in arenas (e.g. Krachun *et al.* 2010; Duve *et al.* 2012; Jensen *et al.* 2015) or kept in larger groups and bigger enclosures compared to the hutches used in the current study (e.g. home pen sizes of 7.08 × 4.74 m [length × width] for Rushen & de Passillé 2012; 17.3 × 3.2 m for Rushen *et al.* 2016). A consideration when using the arena test is that play levels may reflect not only background affective state but also depend on the home pen environment (Jensen & Kyhn 2000). Although our hutches were larger than the home pens in Jensen and Kyhn (2000) that were described to increase locomotor play in an arena, they had a narrow aperture separating the indoor and outdoor spaces, which made it impossible for calves to run between the indoor and outdoor areas. As such, play restriction in the hutches may have caused some motivational rebound of play behaviour in the arena, partially masking treatment differences relating to reduced energy intake and/or negative affective state. Although measuring spontaneous locomotor play in large home pen environments may be subject to fewer confounds (Held, in press), the arena test is useful in situations when measuring home pen play is not feasible. In terms of other potential issues, arena play behaviour in calves can vary from day-to-day (e.g. Mintline *et al.* 2012), and average play levels across multiple test days were not assessed here.

Despite these limitations, our results show that milk allowances affected play responsiveness more strongly than absolute locomotor play levels. Specifically, standard-fed calves were less likely to respond to another calf playing by joining in and playing themselves, despite showing similar numbers of locomotor play bouts overall. It should be remembered that hunger involves components of both affective state and nutrition, but a potential explanation for this result is that when calves are experiencing more negative affect (i.e. due to hunger in this context), their ‘threshold’ for joining in with play behaviour may be higher. As such, for feed-restricted calves, the stimulus of entering a larger space may still elicit play, but observing another calf playing may be less effective at doing so.

Notably, these results suggest that certain characteristics of play behaviour may be more sensitive than others to differences in hunger and affective state in general. For instance, while calves in a more negative affective state (i.e. the feed-restricted calves) may retain their motivation to engage in a certain amount of locomotor play, their interest in more social forms of play, such as running around with another calf (referred to as parallel locomotor play by Bailly-Caumette *et al.* 2023) may be dampened compared to their enhanced-fed counterparts. Indeed, social and locomotor forms of play in calves have been suggested to be at least partially differentially motivated (e.g. Bertelsen & Jensen 2019; Sutherland *et al.* 2019), meaning they could be affected by calves’ experiences in different ways (see Held, in press). In future research, it would be useful to determine whether these play behaviours respond in similar ways to other situations that may impact affective state without significantly involving nutrition (e.g. positive emotion inductions; disbudding, a common but painful husbandry procedure; Mintline *et al.* 2013; Marquette *et al.* 2023; St John Wallis *et al.* 2025a).

### Animal welfare implications

The results of this study have implications for animal welfare assessments. Specifically, in studies using play behaviour to assess welfare, play responsiveness should be explored further as a potentially more sensitive indicator of calves’ experiences compared to absolute play levels, especially when home pen play measurements are not feasible and the arena test is used. Assessing a combination of play variables across the spectrum of locomotor play, social play, and the dynamics of parallel locomotor play behaviour may allow for more accurate and comprehensive assessments of calves’ experiences. Indeed, if play responsiveness had not been assessed in this work, no effect of milk allowance on any play behaviour in the arena would have been identified. In studies focusing on the impact of treatments such as different milk allowances on calves’ welfare, such results could lead to potentially misleading conclusions.

These findings also highlight the need to explore and mitigate the way in which commercial systems affect calves’ ability to move and play. Specifically, the observed changes in play dynamics suggest a potential impact of standard milk allowances on calves’ propensity to play within their social groups (see Größbacher *et al.* 2020). Play is an important behaviour, as it not only indicates but also contributes to affective state and welfare, meaning restrictions on play behaviour may have significant future consequences for animals. For example, play behaviour is known to be rewarding and pleasurable, as shown in rats (Trezza *et al.* 2010; Rygula *et al.* 2012). Play behaviour in juveniles has also been suggested to be important for the development of sociosexual behaviour (e.g. Ahloy Dallaire & Mason 2017), social skills, and likely executive functions (Pellis *et al.* 2014; Pellis & Pellis 2017), behavioural flexibility, and resilience (Špinka *et al.* 2001; St John Wallis *et al.* 2025b), which are important factors in maintaining good welfare in response to the challenges faced by captive individuals (Colditz 2022).

## Conclusion

This work aimed to examine how various aspects of play, including less studied behaviours such as play responsiveness, are affected by milk allowances in calves and, thus, further explore the use of play behaviour as a welfare indicator. The effects of a standard vs enhanced milk allowance on the absolute levels of locomotor play behaviours were limited. However, standard-fed calves were less responsive to locomotor play in their companion calf, meaning they were less likely to join in with the other’s locomotor play bouts. Potentially, calves experiencing more negative affect due to hunger may show lower play responsiveness due to them having a higher ‘threshold’ for play behaviour being elicited or reduced motivation to engage in parallel but not individual locomotor play behaviour. These findings highlight that variables capturing the social dynamics of play behaviour, such as play responsiveness, may provide a more sensitive indicator of a calf’s affective state and welfare compared to absolute locomotor play levels and, thus, warrant further research, especially when play behaviour is measured in an arena test.

## Supporting information

10.1017/awf.2026.10091.sm001 St John Wallis et al. supplementary material St John Wallis et al. supplementary material

## Supplementary material

The supplementary material for this article can be found at http://doi.org/10.1017/awf.2026.10091.

## Table 1. Long description

The table has two columns: Behaviour and Description. From top to bottom, the behaviours are Running, Buck, Kick, Play fighting, and Play initiation and play joining (paired test). Running is described as the calf trotting, cantering, or galloping, with run bouts separated by three seconds or more counted as different bouts; pauses less than three seconds continue the same bout. Buck is when the calf kicks both hind legs up or back. Kick is when the calf kicks one hind leg up, back, or sideways. Play fighting involves two calves pushing their heads together or one pushing its head against another. Play initiation and play joining (paired test) is when one calf starts running and another joins at the same time, or within three seconds of the first finishing.

Navigate back to Table 1..

## Figure 2. Long description

Panel A at the top shows run duration in seconds on the y axis and test type on the x axis. Enhanced-fed calves have higher mean run duration than standard-fed calves in both individual and paired tests, with paired tests showing the highest values for both groups. Panel B in the middle shows run bout frequency on the y axis and test type on the x axis. Both feeding groups have similar run bout frequencies, with slightly higher means in the paired condition. Panel C at the bottom shows point play frequency on the y axis and test type on the x axis. Both feeding groups show similar point play frequencies in individual and paired tests. Error bars represent 95 percent confidence intervals. The legend at the right of each panel identifies enhanced-fed calves in magenta and standard-fed calves in green.

Navigate back to Figure 2..
